# Outcomes of an Emergency Department opioid alternatives Program implemented within a safety-net hospital system

**DOI:** 10.1186/s12873-024-01168-7

**Published:** 2025-01-08

**Authors:** Magda Montague, Charlotte Hopson, Claire Layton, Jennifer Fishe, Ashley Norse, L. Kendall Webb, Petra Duran-Gehring, Andrew Bertrand, Jennifer Brailsford, Taylor Munson, Rui Wang, Nolan Menze, Katelyn Perl, Phyllis Hendry, Sophia Sheikh

**Affiliations:** 1 Department of Emergency Medicine, University of Florida College of Medicine-Jacksonville, 655 West 8th Street, Jacksonville, FL 32209 USA; 2 Center for Data Solutions, University of Florida College of Medicine-Jacksonville, Jacksonville, FL USA; 3 Integrated Data Repository, University of Florida, Gainesville, FL USA

**Keywords:** Opioid alternative, Pain management, EMR order panels, Non-opioid analgesic

## Abstract

**Background:**

The ongoing opioid epidemic in the United States has reinforced the need to provide multimodal and non-opioid pain management interventions. The PAMI-ED ALT program employed a multifaceted approach in the Emergency Department (ED) developing electronic health record (EHR) pain management order panels and discharge panels, as well as educating patients, clinicians, and ED staff on opioid alternatives, including non-pharmacologic interventions. The primary objective of this analysis was to compare changes in opioid and non-opioid analgesic administrations and prescribing in ED patients with select pain conditions (renal colic, headache, low back, and non-low back musculoskeletal pain) before and after implementation of PAMI ED-ALT. Secondary outcomes included characterizing changes in 30-day ED all-cause recidivism and hospital all-cause admissions within these pain populations.

**Methods:**

Demographics, opioid and opioid alternative utilization, hospital admission, 30-day ED returns and change in pain intensity score were collected from January 2019-March 2020 (pre-program implementation) and January 2021-March 2023 (post-program implementation) for both the ED aggregate and program target pain populations.

**Results:**

Pain management order panel utilization increased throughout the post-implementation period. When comparing pre to post program data, there was a reduction in opioid administrations and prescriptions for most of the target pain conditions, as well as within the ED aggregate population. Opioid alternative administrations and prescriptions increased for all pain conditions except renal colic. Hospital admissions decreased significantly amongst those with low back pain and headache/migraine and 30-day ED returns significantly declined in those with musculoskeletal pain.

**Conclusion:**

Our findings demonstrate an opioid-alternatives program implemented within a safety-net hospital system serving a predominantly socially disadvantaged patient population can lead to changes in ED pain management and potentially reduce 30-day ED recidivism and hospitalizations.

**Supplementary Information:**

The online version contains supplementary material available at 10.1186/s12873-024-01168-7.

## Background

Pain is one of the most common reasons for presenting to the Emergency Department (ED) [1, 2]. Patients often seek care in the ED for acute pain, including acute exacerbations of an underlying medical condition, particularly when unable to manage their pain at home or within other healthcare settings. In the late 1990s and early 2000s, there was a shift towards opioid-based pain management that was not specific to the ED setting but permeated all healthcare settings [3]. While, ED clinicians are not among the top opioid prescribers in the United States (and in fact ED opioid prescribing rates had reportedly declined and stabilized by 2012), because of the nature of the types of patients ED clinicians treat (traumatic injuries, severe exacerbations of comorbid conditions, etc.), opioid medications are an important part of ED pain management [4, 5]. Thus, it is for this reason that ED clinicians must also be good stewards of opioid-based pain management and utilize multimodal approaches when possible. However, there are limited evidence-based opioid-prescribing guidelines relevant to ED clinicians that provide guidance on multimodal approaches [3].

The ongoing opioid epidemic in the United States has reinforced the need to provide multimodal and non-opioid pain management interventions. As a consequence of the opioid epidemic, a plethora of mandatory education has focused on improving opioid stewardship and recognizing the links between pain, opioid medication initiation and development of long term opioid use and opioid use disorder [6–8]. Until recently, most ED clinicians and staff had limited to no education and training on multimodal pain management plans, particularly non-pharmacologic techniques [9, 10]. However, simply reducing opioid prescriptions alone falls short of addressing the underlying reason for the opioid prescription. Effective pain management must address the factors contributing to pain while also balancing patient safety and quality of life. New strategies must therefore focus on non-opioid pain management (alone or as an adjunct, including non-pharmacologic techniques) to treat pain and improve outcomes, while also decreasing opioid use and potential for abuse.

To address this need, the Pain Assessment and Management Initiative ED Alternatives to Opioids (PAMI-ED ALT) program was created. Similar opioid stewardship programs have also been implemented across the country in the wake of (and frankly ongoing) opioid epidemic, but few have published their outcomes [11]. PAMI-ED ALT employed a multifaceted approach developing electronic health record (EHR) pain management order panels and discharge panels, as well as educating patients, clinicians, and ED staff on opioid alternatives, including non-pharmacologic interventions. The program focused on enhancing current clinical operations and utilizing implementation science and the Diffusion of Innovation Theory (adoption or diffusion of new ideas or behaviors in a population over time) to create sustained changes in ED prescribing practices related to opioid stewardship and pain management [12, 13]. Program interventions were designed to promote multimodal pain management and improve opioid stewardship by automating and streamlining evidence-based pain management within the EHR, performing targeted clinician and patient education, and reinforcing program efforts through an implementation-outcomes feedback loop. Previous studies have found integration of order panels into the EHR significantly improved the overall adoption of those orders by clinicians; while leveraging implementation science and the Diffusion of Innovation Theory are key to translating knowledge into clinical practice [12–17].

The primary objective of this analysis was to compare changes in opioid and non-opioid analgesic administrations and prescribing in ED patients with select pain conditions before and after implementation of PAMI ED-ALT. Secondary outcomes included characterizing changes in 30-day ED all-cause recidivism and hospital all-cause admissions within these pain populations.

### Methods

#### Study setting

University of Florida Health Jacksonville (UF Health Jacksonville) healthcare system includes an academic urban safety-net hospital system and Level 1 adult and pediatric trauma center caring for a predominantly socioeconomically disadvantaged population. The healthcare system is a public not-for-profit entity contracted with the city to provide care for the uninsured. It has 695 beds, with a combined annual volume of approximately 23,685 admissions and 103,760 ED visits. The average ED patient is 47 years old, 52% self-report as African American, 94% as non-Hispanic, 44% are on Medicare or Medicaid, 33% on commercial insurance, and 23% are self-pay or receive city healthcare assistance. The mean Area Deprivation Index national percentile for adult ED patients presenting for pain to UF Health Jacksonville is 83 (scores range from 0–100, higher percentiles indicate higher disadvantage) [18]. During the study period, the ED was staffed by 47 attending physicians, 52 ED physician-trainees (residents and fellows), 21 advanced practice providers, and > 300 nurses, technicians, paramedics, and patient care assistants. Since data was collected from the entire ED population presenting during the study period and individual patient enrollment did not occur, the project was deemed a quality improvement project by the University of Florida Institutional Review Board. It is registered within the University of Florida’s Quality Improvement Project Registry (Project #1362). The funding organizations and sponsors had no role in the development, conduct, or reporting of the study.

#### Program details

In January 2021, the PAMI ED-ALT program launched a new set of pain management order panels in the EHR for the treatment of four specific pain conditions: renal colic, headache, low back, and non-low back musculoskeletal pain. While the order panels were intended specifically for these pain conditions, there were no restrictions or verification requirements for use by clinicians. The pain management order panels were added to the institution’s Epic “Quick List” (highly visible order panels that appear when clinicians place orders) for the ED to facilitate clinician access and ease of use. The program focused on common sources of pain for which opioid alternative evidence-based pain management plans were available [19–25]. The order panels contained both pharmacologic (opioid and non-opioid) and non-pharmacologic treatment options (lavender aromatherapy, hot/cold packs, hand acupressure device targeting the LI4 pressure point, pain journaling, coloring and virtual reality) to integrate opioid alternatives into ED clinician’s workflows (Supplemental Materials Fig. 1) [19, 26–29]. Additionally, links to educational resources, including “how-to” videos for pain management procedures, such as nerve blocks and trigger point injections, were linked within the EHR order panels. Supplies for non-pharmacologic orders were kept in auto-locking carts throughout the clinical areas for ED clinicians and staff to access.

Program champions were utilized to help promote program initiatives and were selected for the role through a peer and self-nomination process after a detailed description of the role was distributed to ED staff. Program champions included three nurses, two advanced practice providers, one emergency department physical therapist and two emergency medicine resident physicians. Champions were vital in promoting project initiatives to fellow staff members during staff meetings, nursing huddles and served as a liaison between project staff and their clinical colleagues.

Education on the non-pharmacologic supplies and order panels were regularly provided to ED providers throughout the life of the project to promote utilization and adoption (e.g., formal in-person training, frequent reminders/updates in weekly ED operations emails and calls) and were reinforced through an implementation-outcomes feedback loop. The feedback loop consisted of sharing monthly utilization and program metrics with ED clinicians via a “dashboard” to increase awareness and project buy-in and to identify barriers and enablers to widespread dissemination of program efforts (see Supplemental Materials Figs. 2 and 3). Direct feedback on utilization was also provided to clinicians by recognizing top utilizers for the month. Top program utilizers were recognized as “PAMI ED-ALT Star of the Month”. Two clinicians were selected each month and they received a certificate of appreciation, a thank-you goody bag, and were highlighted on a bulletin board in the ED hallway.

#### Variables collected

Utilization metrics for the four pain management order panels were collected over the study period (January 2019 – March 2023). Patients presenting with select target pain conditions were identified using the International Classification of Diseases, Tenth Revision, Procedure Coding System, Clinical Modification (ICD10-CM) codes described in Supplemental Materials [30]. The following variables were collected at both the ED aggregate level (i.e. entire adult ED population) and for the program target population (i.e. those patients presenting to the ED with one of four target pain conditions- renal colic, headache, low back, and non-low back musculoskeletal pain): demographics (race, age, ethnicity, gender); opioid medication (codeine, fentanyl, morphine, hydrocodone, hydromorphone, oxycodone, tramadol) administrations and prescriptions (administrations, opioid medications given during the ED encounter; prescriptions, opioid medications prescribed at ED discharge); opioid alternative administrations and prescriptions (oral or topical non-steroidal anti-inflammatory drugs [NSAIDs], acetaminophen, topical lidocaine, gabapentin, muscle relaxers [cyclobenzaprine, methocarbamol, diazepam, and tizanidine], and butalbital/acetaminophen/caffeine); change in pain intensity score (from time of ED triage to ED discharge); and hospital admission (yes/no) and ED returns for any reason within 30 days (yes/no) as determined by review of the EHR. Emergency Severity Index (ESI) acuity classification, and ED trauma activation volume were also collected but only at the ED aggregate level [31].

#### Data analysis

Order panel utilization metrics were grouped into quarters for each year of the study period (January 2019-March 2023). EHR data was extracted from a curated SQL-based clerical warehouse from January 2019-March 2020 (pre-program implementation) and January 2021-March 2023 (post-program implementation). SQL based-databases or warehouses compile a copy of clinical and administrative data from the EHR in near real time facilitating data queries and analyses [32]. The pre-program implementation dates were chosen to mitigate the effects of the COVID-19 pandemic on ED utilization and related ED metrics. Monthly data were aggregated to cover the entire pre-implementation period and the entire post-implementation period for both the ED aggregate population and the program target pain population. Demographic and 30-day ED revisit comparisons between implementation periods were performed at the unique patient level. As the overwhelming majority of the study population (> 99%) was comprised of patients who self-reported as African American or White, only these races were examined in the data analysis. ESI emergent, immediate and urgent acuity classification levels were combined as one category (termed high acuity) and compared to a combined categorization of ESI less urgent and non-urgent (termed low acuity). Proportional variables for the pre- and post-implementation periods were compared using Chi-square tests. Continuous variables were compared using Wilcoxon Rank Sum or unpaired t-test with significance measured at *p* < 0.05. Descriptive statistics were analyzed using Excel 2016. Statistical comparisons were conducted in R (4.1.2) 2021.

## Results

### Pain management panel utilization

**Fig. 1:**
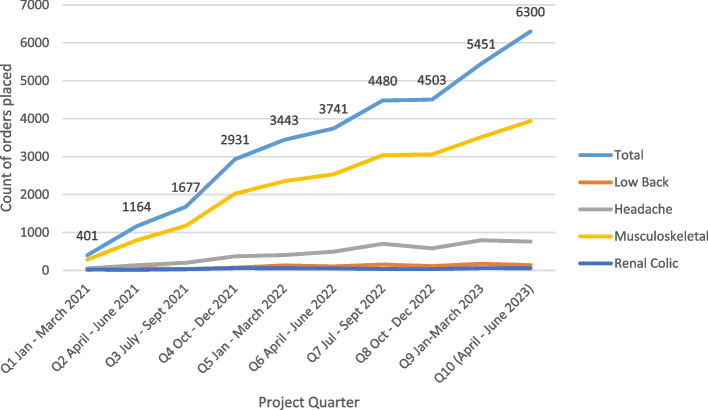
Quarterly panel utilization since program launch

Figure 1 depicts quarterly panel utilization since program launch. Total panel utilization increased every quarter, with a slight reduction in Quarter 8 (October 2022-December 2022). The majority of orders from the pain management panels were for treatment of patients with musculoskeletal pain.

### ED demographics

Table 1 compares the demographic characteristics of the aggregate ED population and program target population during the pre- and post-program implementation periods. Mean age, gender, and ethnicity were distributed similarly for both groups between implementation periods. There were significantly less patients in the target population self-reporting as White during the post-implementation period compared to the pre-implementation period (*p* = 0.008). The distribution of race was not significantly different between the periods for the aggregate ED population. The number of unique patients in which the order panels were utilized increased throughout the post-implementation period (Supplementary Fig. 4). Trauma activations increased across periods (pre-period 5.5% versus post-period 6.4%, *p* < 0.001) as did ESI acuity levels (high acuity- pre-period 74% versus post-period 76%; low acuity- pre-period 19% versus post-period 16%, *p* < 0.001).

**Table 1:** ED aggregate and program target pain populations demographic comparisons

	ED Aggregate Population			Program Target Population		
	Pre – Program (N,%) ^a^(*N* = 134,870; 117,668)	Post – Program (N,%) ^a^(*N* = 237,001; 203,131)	*p*-value	Pre – Program (N,%) ^a^(*N* = 67,354; 55,427)	Post -Program (N,%) ^a^(*N* = 92,924; 79,473)	*p*-value
**Gender**						
Female	(64,418) 54.7%	(109,257) 53.8%	** < 0.001**	(30,833) 55.6%	(43,290) 54.5%	** < 0.001**
Male	(53,153) 45.2%	(93,544) 46.1%	** < 0.001**	(24,606) 44.4%	(36,059) 45.4%	** < 0.001**
**Race**						
Black	(63,043) 53.6%	(107,386) 52.9%	0.128	(29,035) 52.4%	(41,598) 52.3%	**0.008**
White	(46,900) 39.9%	(78,955) 38.9%	0.128	(23,182) 41.8%	(32,216) 40.5%	**0.008**
Asian	(598) 0.5%	(1,263) 0.6%		(258) 0.5%	(493) 0.6%	
Multi-Racial	(464) 0.4%	(1,022) 0.5%		(208) 0.4%	(400) 0.5%	
Other	(5,454) 4.6%	(10,511) 5.2%		(2,443) 4.4%	(3,869) 4.9%	
Unknown	(1,209) 1.0%	(3,994) 2.0%		(301) 0.5%	(897) 1.1%	
Age* (mean)	45.8	46.6	** < 0.001**	48.5	48.9	**0.005**
**Ethnicity**						
Non-Hispanic	(111,283) 94.6%	(188,778) 92.9%	** < 0.001**	(52,791) 95.2%	(73,978) 93.1%	** < 0.001**
Hispanic	(4,837) 4.1%	(10,039) 4.9%	** < 0.001**	(2,219) 4.0%	(3,707) 4.7%	** < 0.001**
**Insurance**^**b**^			** < 0.001**			** < 0.001**
Medicaid	(25,385) 20.9%	(44,475) 21.8%		(10,849) 19.6%	(15,576)19.7%	
Medicare	(26,656) 21.0%	(46,490) 22.8%		(14,037) 25.3%	(20,000) 25.3%	
Charity	(25,346) 20.9%	(26,146) 12.8%		(10,775) 19.4%	(9,906) 12.6%	
Self-pay	(10,232) 8.4%	(21,928) 10.8%		(3,185) 5.7%	(5,761) 7.3%	
Commercial	(33,684) 27.7%	(64,148) 31.5%		(16,514) 29.8%	(27,428) 34.8%	
Other	(215) 0.2%	(393) 0.2%		(80) 0.1%	(232) 0.3%	
**Change in pain score (mean)**^**c**^	−2.3	−2.6	** < 0.001**	−2.7	−3.1	** < 0.001**

*P*-value from chi-square test with continuity correction except where designated * which are Wilcoxon Rank Sum Tests

^a^(N = Total ED volume; number of unique patients)

^b^More than one insurance category may be possible

^c^Denominator used is unique number of patients. Comparison test by unpaired t-test

### Program target population

#### Opioid-alternative medications

Table 2 compares pre- and post- program data for the program target population by pain type. ED administrations and prescriptions of opioid-alternative medications significantly *increased* in the post-implementation period compared to the pre-implementation period for all pain types (*p* < 0.05), except renal colic. For renal colic, opioid-alternative prescriptions fell nearly 5% (*p* = 0.001) and ED administrations remained unchanged.

#### Opioid medications

Across implementation periods, ED administrations of opioid medications significantly *decreased* in the program target population for low back pain and headache/migraine (*p* < 0.001) (Table 2). ED opioid administrations *increased* by 1.5% for musculoskeletal pain. ED prescriptions for opioid medications decreased in patients presenting with headache/migraine (*p* = 0.015) and renal colic (*p* = 0.007) over the implementation periods.

#### Non-pharmacologic interventions

The most common items used during the post-implementation period were the hot/cold packs (8299) followed by aromatherapy inhalers (5127), pain journals (1320), virtual reality (1022), coloring sheets (857) and acupressure devices (735). Supplementary Fig. 5 depicts quarterly distribution of non-pharmacologic items.

#### Change in Pain Scores

There was a greater reduction in pain scores during the post-implementation period than during the pre-period (*p* < 0.001) (Table 1).

#### Hospital all-cause admissions data

Hospital admissions among patients with low back pain and headache/migraine decreased significantly (*p* < 0.001) across implementation periods (Table 2).

#### 30-day all-cause ED returns

Revisits among patients with renal colic, musculoskeletal, and headache/migraine decreased, but only reductions among patients with musculoskeletal pain were significant (*p* < 0.001) (Table 2).

**Table 2:** Comparison of pre- and post- program data for the program target population by pain type

	**Pre – Program (N, %)** ^a^(*N* = 67,354; 55,427)	**Post -Program (N, %)** ^a^ (*N* = 92,924; 79,473)	^**b**^**Change**	***p*-value**
**Low back pain**	**(*n*= 1,153)**	**(*n* = 1,898)**		
Opioid Alternative Administrations	(771) 66.9%	(1,341) 70.7%	3.8%	**0.031**
Opioid Alternative Prescriptions	(493) 42.8%	(913) 48.1%	5.3%	**0.005**
Opioid Administrations	(486) 42.2%	(659) 34.7%	−7.4%	** < 0.001**
Opioid Prescriptions	(110) 9.5%	(164) 8.6%	−0.9%	0.437
Hospital all-cause admissions	(455) 39.5%	(615) 32.4%	−7.1%	** < 0.001**
ED 30-day all-cause revisits^**c**^	(225) 19.5%	(393) 20.7%	1.2%	0.455
**Headache/migraine**	**(*n* = 11,254)**	**(*n* = 16,602)**		
Opioid Alternative Administrations	(6,502) 57.8%	(10,439) 62.9%	5.1%	** < 0.001**
Opioid Alternative Prescriptions	(1,728) 15.4%	(2,798) 16.9%	1.5%	**0.001**
Opioid Administrations	(1,834) 16.3%	(2,438) 14.7%	−1.6%	** < 0.001**
Opioid Prescriptions	(289) 2.6%	(351) 2.1%	−0.5%	**0.015**
Hospital all-cause admissions	(2,447) 21.7%	(3,080) 18.6%	−3.2%	** < 0.001**
ED 30-day all-cause revisits^**c**^	(2,404) 21.4%	(3,486) 21.0%	−0.4%	0.475
**Musculoskeletal pain**	**(***n*** = 53,426)**	**(***n*** = 71,856)**		
Opioid Alternative Administrations	(21,903) 41.0%	(36,666) 51.0%	10.0%	** < 0.001**
Opioid Alternative Prescriptions	(10,750) 20.1%	(17,247) 24.0%	3.9%	** < 0.001**
Opioid Administrations	(14,065) 26.3%	(20,004) 27.8%	1.5%	** < 0.001**
Opioid Prescriptions	(3,127) 5.9%	(4,151) 5.8%	−0.1%	0.577
Hospital all-cause admissions	(13,852) 25.9%	(18,694) 26.0%	0.1%	0.729
ED 30-day all-cause revisits^**c**^	(12,345) 23.1%	(15,978) 22.2%	−0.9%	** < 0.001**
**Renal colic**	**(***n*** = 1,521)**	**(***n*** = 2,568)**		
Opioid Alternative Administrations	(949) 62.4%	(1,627) 63.4%	1.0%	0.560
Opioid Alternative Prescriptions	(415) 27.3%	(580) 22.6%	−4.7%	**0.001**
Opioid Administrations	(809) 53.2%	(1,318) 51.3%	−1.9%	0.262
Opioid Prescriptions	(456) 30.0%	(669) 26.1%	−3.9%	**0.007**
Hospital all-cause admissions	(570) 37.5%	(959) 37.3%	−0.1%	0.960
ED 30-day all-cause revisits^**c**^	(342) 22.5%	(565) 22.0%	−0.5%	0.748

*P*-value from chi-square test with continuity correction except where designated * which are Wilcoxon Rank Sum Tests

^a^(N = Total ED volume; number of unique patients)

^b^Change between program periods

^c^Denominator used is unique number of patients. Opioid administrations, opioid medications given during the ED encounter. Opioid prescriptions, opioid medications prescribed at ED discharge

### ED aggregate population

#### Opioid-alternative medications

 Across implementation periods, ED administrations of opioid alternative medications increased (*p* < 0.001) but ED prescriptions decreased (*p* < 0.001) (Table 3).

**Table 3:** Comparison of pre and post program data for the ED aggregate population

	Pre – Program (N, %) ^a^(*N* = 134,870; 117,668)	Post – Program (N, %) ^a^(*N* = 237,001; 203,131)	^b﻿^Change	*p*-value
Opioid Alternative Administrations	(42,877) 31.8%	(79,284) 33.5%	1.7%	< 0.001
Opioid Alternative Prescriptions	(17,104) 12.7%	(27,437) 11.6%	−1.1%	< 0.001
Opioid Administrations	(26,373) 19.6%	(42,732) 18.0%	−1.5%	< 0.001
Opioid Prescriptions	(5,813) 4.3%	(8,316) 3.5%	−0.8%	< 0.001
Change in pain score (mean)*	−2.3	−2.6	−0.3	< 0.001
Hospital all-cause admissions	(33,057) 24.5%	(55,535) 23.4%	−1.1%	< 0.001
ED 30-day all-cause revisits^c^	(13,666) 11.6%	(23,426) 11.5%	−0.1%	0.49

*P*-value from chi-square test with continuity correction except where designated * which are Wilcoxon Rank Sum Tests.

a(*N* = Total ED volume; number of unique patients)

^b^Change between program periods

^c^Denominator used is unique number of patients. Opioid administrations, opioid medications given during the ED encounter. Opioid prescriptions, opioid medications prescribed at ED discharge

#### Opioid medications

ED opioid medication administrations and prescriptions both declined from the pre- to the post-implementation periods (*p* < 0.001) (Table 3).

#### Change in Pain Scores

There was a greater reduction in pain scores during the post-implementation period than during the pre-period (*p* < 0.001) (Table 1).

#### Hospital all-cause admissions data

 Hospital admissions decreased in the post-implementation period (*p* < 0.001) (Table 3).

#### 30-day all-cause ED returns

Revisits among the aggregate population did not change over the implementation periods (*p* = 0.49).

## Discussion

The objective of this analysis was to characterize the impact of the PAMI-ED-ALT program on ED administration and prescription of opioids and opioid alternatives in patients presenting to the ED for renal colic, headache, low back pain, musculoskeletal pain. When comparing our pre- and post-program data, we found a reduction in opioid administrations and prescriptions in most pain conditions and observed a reduction for both variables in the ED aggregate population. Opioid alternative administrations and prescriptions increased for all pain conditions except renal colic. A similar, but not as substantial, increase in opioid alternative administrations was also observed in the ED aggregate population. The secondary objective was to assess impact on hospitalizations and ED recidivism. Hospital admissions decreased significantly amongst those with low back pain and headache/migraine, and 30-day returns declined in those with musculoskeletal pain. It is worth noting that we observed a greater reduction in pain scores during the post- compared to the pre-implementation period.

Panel utilization increased throughout the post-implementation period. There were notable spikes during quarters 3 and 8 after changes to the EHR user interface were made to the pain management order panels based on clinician feedback. One example of such feedback included suggestion of creating a “Quick List” within the institution’s EHR to increase visibility of the order panels and limit time searching for the panels. Existing literature demonstrates successful adoption by clinicians of disease-specific order subsets when they were integrated within general prescribing ordersets [17]. Additionally, clinician education, via formal and informal trainings, occurred twice per year during the study period; involvement of program champions and buy-in from institutional and ED leadership; along with dissemination of monthly “dashboards” communicating program metrics to ED clinicians and staff were other key factors in driving adoption and utilization of the panels into routine practice.

Within healthcare, the Diffusion of Innovation Theory has accelerated the adoption of programs aiming to change the behavior of a social system through development of stepwise interventions and performance assessments. Those steps include developing an intervention to address a specified problem, promoting the intervention (persuasion), having adopters decide to try the intervention, implementation of the intervention at scale, and confirmation of success [12–16, 33–40]. We used the Diffusion of Innovation theory to conceptualize adoption and integration of multimodal pain management into emergency medicine practice [13, 16]. We created a continuous feedback loop to promote program initiatives (educational sessions and program champions), identify areas for improvement (clinician and champion feedback), communicate performance metrics to the project team and ED clinicians (monthly dashboard), and measure programmatic success and adoption by providing evidence of improved patient care and outcomes (opioid and non-opioid medication prescribing, non-pharmacologic modalities, 30-day ED returns and hospitalizations).

There were slight demographic changes (race, gender, ethnicity) in the program target group when comparing pre- and post-implementation periods. Some of these changes were also seen in the overall ED aggregate population as well. Additionally, there were changes in insurance coverage across implementation periods within the target group that mirrored changes seen in the overall aggregate ED population, with a higher proportion of patients reporting self-pay status and commercial coverage and fewer reporting charity/city-funded coverage in the post-implementation period. Although there were statistically significant changes in many demographic variables, the overall composition of the program target population did not change, with the population still being predominantly female, non-Hispanic, and Black. Thus, it is unlikely that demographic factors contributed significantly to differences in outcomes between the pre- and post-implementation periods.

Within our program target population, the use of opioid-alternative medications during the ED encounter increased post-implementation for all pain types, except renal colic (unchanged). Despite an overall reduction in prescribing of opioid-alternatives at the ED aggregate level (−1%), prescriptions for opioid-alternative medications actually *increased* in all target pain types, except renal colic where prescriptions decreased by 5%. It is unclear why opioid-alternative prescriptions in patients with renal colic decreased when administrations during the ED encounter itself remained unchanged. As NSAIDS are typically first line treatment for renal colic and there are many formulations available without a prescription, it is possible clinicians may have changed practice during the post-implementation period (potentially in response to program education on readily available over-the-counter analgesics) and were now instructing patients to use over-the-counter opioid-alternatives at home without writing prescriptions, leading to the appearance of a reduction in opioid alternative prescribing.

We observed ED opioid administrations and prescriptions within our aggregate ED population significantly declined over the implementation periods (−1.5%). The cause of this overall decline may be attributed to multiple factors, including increased clinician awareness of the opioid epidemic and potential harms associated with opioid prescribing (evidenced by opioid prescribing rates declining nationally), as well as a diffusion of the program to other pain populations (“diffusion effect”). Clinicians influenced by the program may have utilized program principles, resources, and materials (i.e. knowledge gained from educational sessions, pain management order panels, non-pharmacologic supplies, etc.) when treating populations that were not within the intended scope of the program (ED patients presenting for pain complaints not related to the four target conditions) leading to a decline in overall opioid utilization by our ED.

Opioid administrations in patients presenting specifically with headache/migraine and renal colic also significantly declined over the implementation periods and at a slightly higher rate than that of the general ED population (−1.6 and −1.9% versus −1.5%, respectively). ED opioid administrations for the treatment of low back pain declined five-fold across implementation periods compared to the general decline seen in opioid administrations for the overall ED population (−7.4% versus −0.8%), opioid administration rates for musculoskeletal pain *increased* by 1.5%. This increase in opioid administrations may be related to the increase in trauma activations and higher ESI acuity that was seen in the ED during the post-implementation period compared to the pre-period. Thus, there may have been more patients with traumatic injuries necessitating treatment with opioid medications in the ED during the post-implementation period. Others have reported also seeing an increase in opioid administrations in patients with traumatic injuries post-implementation of an opioid alternatives program [41]. However, it is important to note that while opioid administrations during the ED encounter itself increased in those presenting with musculoskeletal pain, our program was not associated with an increase in ED opioid prescriptions at discharge.

We found all-cause 30-day return rates significantly declined across implementation periods in patients presenting with musculoskeletal pain, while no significant change was seen in the other target pain conditions nor the aggregate ED population. There was also a significant reduction in all-cause hospital admissions in patients presenting for low back pain and headache/migraine. While all-cause hospital admissions declined over the periods in the aggregate ED population (−1.1%), the degree of reduction in hospital admissions was greater for low back pain and headache/migraine (−7.1% and −3.2%, respectively). These conditions were also both associated with less opioid medication administrations in the ED post-program implementation and headache/migraine was additionally associated with less opioid prescriptions at ED discharge. There are few, if any, opioid-alternative programs reporting an association with reduced all-cause 30-day ED returns and hospitalizations.

There are several limitations to our study. First, the program was implemented in only one institution. Thus, program results may not be replicable in EDs located in other geographic areas, with differing practice patterns, and populations with different sociodemographic compositions. Second, there are known issues that can arise when extracting EHR data from large databases, including a lack of standardized data formatting and data granularity leading to potential data misinterpretation or discrepancy. For example, we were unable to capture when clinicians may have instructed patients to utilize over-the-counter products as this is not a discrete variable that can be pulled by the EHR analyst and requires manual chart review. Third, given the large scale implementation of this program, individual patient enrollment did not occur thus we were unable to capture the patient perspective. Future qualitative study could include patient semi-structured interviews and/or focus groups to gather this valuable information. Additionally, morphine milligram equivalent or other quantitative measures of opioid data were not collected nor change in pain intensity for the pain target group. However, despite these limitations, our findings demonstrate that an opioid-alternatives program implemented within a safety-net hospital system serving a predominantly socially disadvantaged patient population can lead to changes in ED pain management and potentially reduce 30-day ED recidivism and hospitalizations.

## Supplementary Information

Supplementary Material 1.

Supplementary Material 2.

Supplementary Material 3.

Supplementary Material 4.

Supplementary Material 5.

## Data Availability

The datasets used and/or analyzed during the current study are available from the corresponding author on reasonable request.
