# Supplementary material for: Outcomes of an Emergency Department opioid alternatives Program implemented within a safety-net hospital system
Source: BMC Emerg Med. 2025 Jan 8;25:5. doi: 10.1186/s12873-024-01168-7 (PMC11707854; doi:10.1186/s12873-024-01168-7)

Supplementary Figure 1. Screenshot of pain management order panels for a simulated patient within the EHR

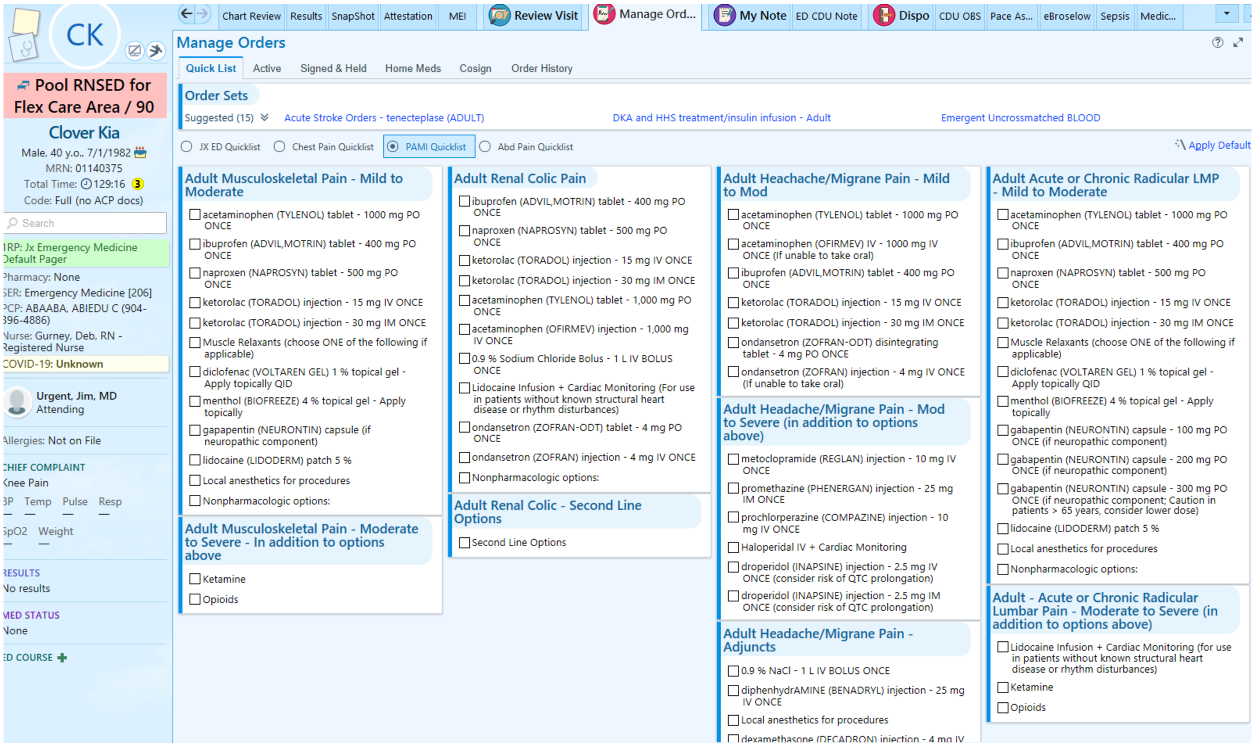

Supplement: Supplementary file 1 — Supplementary Material 1. [file 12873_2024_1168_MOESM1_ESM.pdf]
