# Supplementary figures and images for: Outcomes of an Emergency Department opioid alternatives Program implemented within a safety-net hospital system
Source: BMC Emerg Med. 2025 Jan 8;25:5. doi: 10.1186/s12873-024-01168-7 (PMC11707854; doi:10.1186/s12873-024-01168-7)

Supplementary Figure 2. Dashboard of program metrics

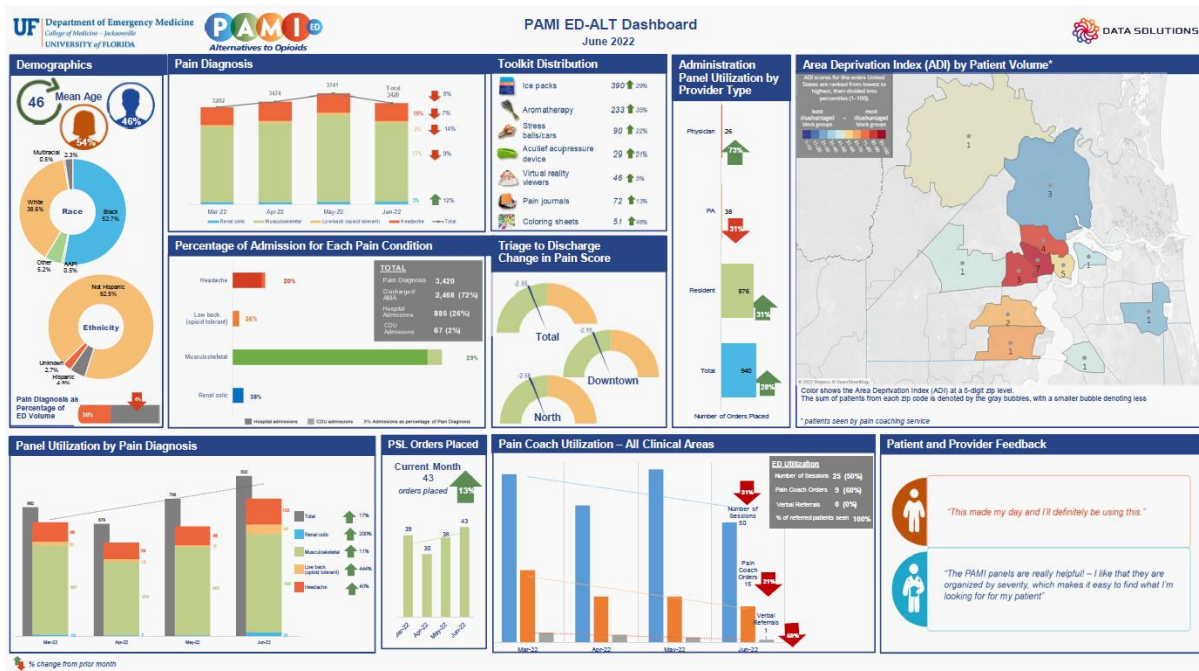

Supplement: Supplementary file 2 — Supplementary Material 2. [file 12873_2024_1168_MOESM2_ESM.pdf]

**Supplementary Figure 4. Quarterly distribution of unique program participants**

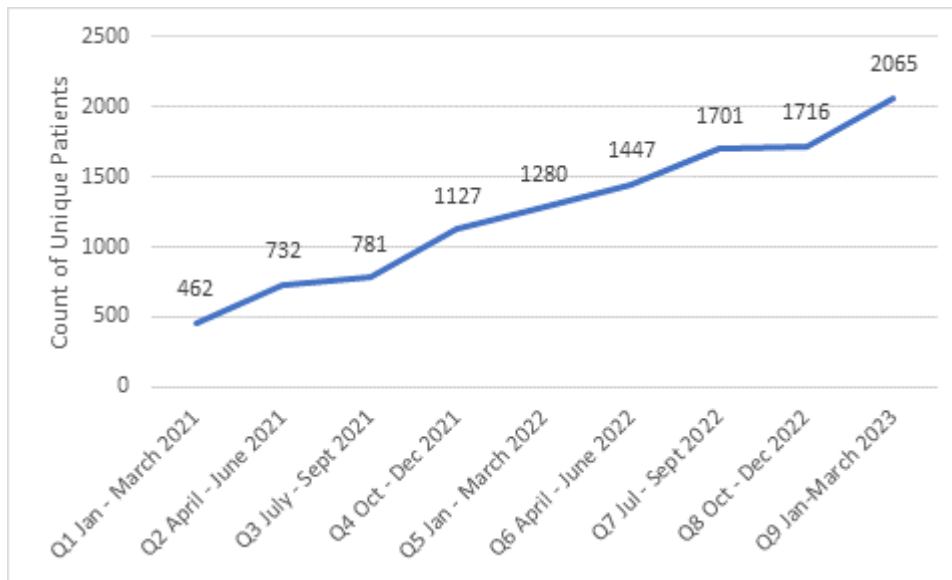

Supplement: Supplementary file 4 — Supplementary Material 4. [file 12873_2024_1168_MOESM4_ESM.pdf]

**Supplementary Figure 5. Quarterly distribution of non-pharmacologic items**

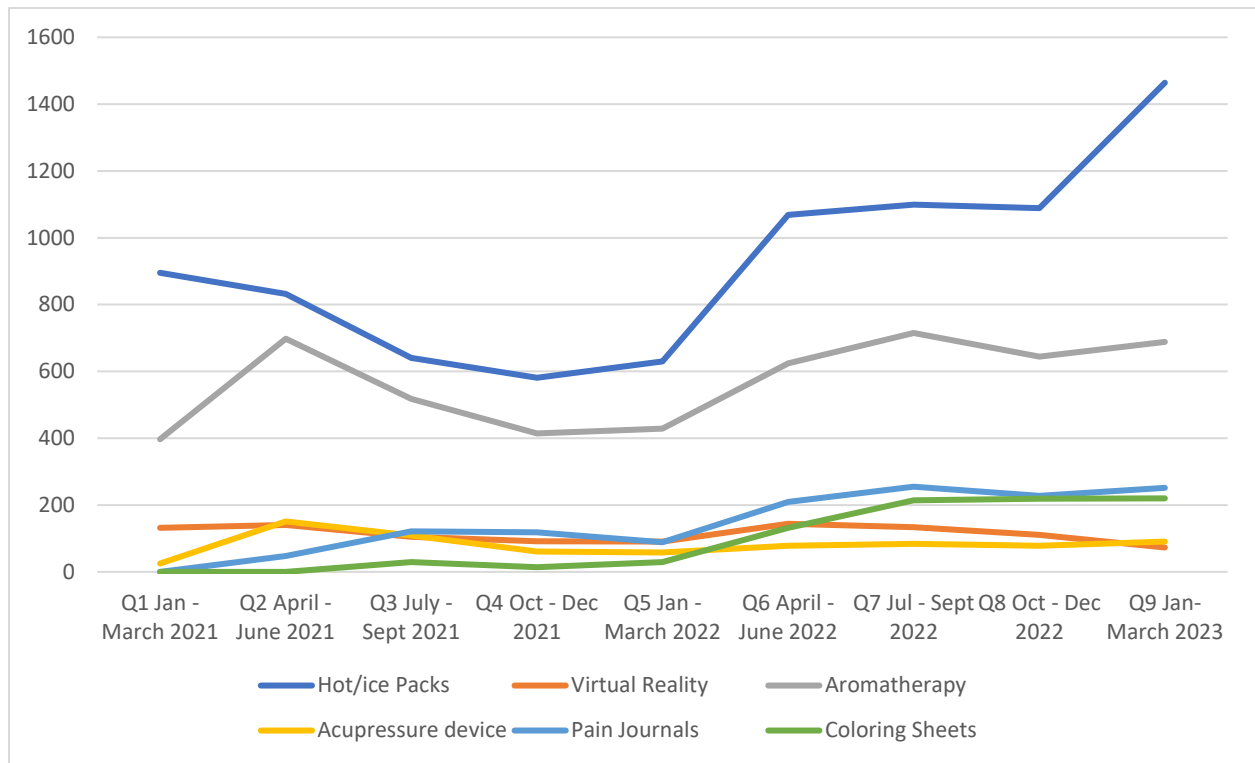

Supplement: Supplementary file 5 — Supplementary Material 5. [file 12873_2024_1168_MOESM5_ESM.pdf]
